# Post-COVID-19 Inflammatory Arthritis Treated With Upadacitinib: A Case Report

**DOI:** 10.7759/cureus.78799

**Published:** 2025-02-09

**Authors:** Cherie S Zhi, Ami Ben-Artzi

**Affiliations:** 1 Rheumatology, Ami Ben-Artzi, MD Inc., Scripps Hospital, San Diego, USA

**Keywords:** auto-immune molecular mimicry, inflammatory arthritis, jak inhibitor, molecular mimicry in covid-19, poly-arthralgia, polyarticular arthritis, post-covid-19, post covid-19 syndrome, upadacitinib, viral arthritis

## Abstract

Two patients who developed inflammatory arthritis following COVID-19 infection are presented, characterized by arthralgias, arthritis, and elevated markers of inflammation (C-reactive protein). Both patients had clinically meaningful responses to treatment with upadacitinib, a Janus Kinase inhibitor (JAKi). This case report highlights the efficacy and safety of upadacitinib in the treatment of post-COVID-19 inflammatory arthritis.

## Introduction

Post-infectious inflammatory arthritis is characterized by joint inflammation following an infectious disease episode. Since the start of the COVID-19 pandemic, there has been a well-documented syndrome of inflammatory arthritis following COVID-19 infection, accompanied by elevated markers of inflammation [1,2]. Patients affected by post-COVID-19 inflammatory arthritis often report persistent diffuse joint pains and fatigue [1]. There is no established association between the severity of COVID-19 infection and arthritis; therefore, even patients with asymptomatic COVID-19 disease can develop severe inflammatory arthritis following the infection. While there is often no detectable synovitis on a physical examination, a subset of patients have tenderness of multiple joints and elevated C-reactive protein (CRP) [1].

Janus Kinase (JAK) is a group of four kinases: JAK1, JAK2, JAK3, and tyrosine kinase 2 (TYK2), which regulate inflammation pathways. One form of such regulation is the JAK-signal transducers and activators of the transcription (STAT) pathway, where the activation of JAK causes STATs to become phosphorylated to enter the nucleus. Once in the nucleus, the STATs activate transcription of inflammatory cascade mediators [3]. There are complex interactions between the various JAKs and cytokine transduction; in general, JAK1 mediates interferon (IFN)-alpha, IFN-beta, and IFN-gamma transduction pathways, JAK3 mediates interleukin (IL)-2, IL-4, IL-7, IL-9, IL-15, and IL-21 transduction pathways, and TYK2 mediates IFN-I, IL-12, and IL-23 transduction pathways [4]. Due to the involvement of these cytokines in autoimmune diseases, the inhibition of JAK using small molecules has been very beneficial for patients with diseases such as rheumatoid arthritis, psoriatic arthritis, and ankylosing spondylitis [5].

Upadacitinib is a JAK inhibitor that suppresses inflammation by competitively inhibiting the binding of adenosine triphosphate (ATP) to JAK, therefore preventing the downstream effects of JAK on inflammation pathways [6]. Both the efficacy and safety of upadacitinib are well documented in clinical trials. Of note, the most common adverse reactions and risks are reactivation of herpes zoster virus (HZV) and upper respiratory infections [6,7]. The severity of these risks can be mitigated via routine follow-ups with the patient for clinical evaluations and laboratory tests (blood counts, liver function, and renal function) [8]. HZV risk can be effectively managed through vaccination and antiviral prophylactic treatment [9].

## Case presentation

Patient A is a 54-year-old female with a medical history significant for a left wrist ulnar ligament injury, which occurred at age 22, and was treated with surgery. After receiving her second COVID-19 vaccine in the left deltoid muscle in May 2021, she developed immediate severe pain and stiffness in the left shoulder, which persisted for a month and then resolved. In February 2022, she received a COVID-19 vaccine booster in the left deltoid muscle, and two months later she developed severe left-side neck pain for a month, which then resolved. 

The patient developed the COVID-19 infection in September 2022. Approximately three weeks later, her left wrist became swollen and very painful, especially at the radial aspect. This was treated with a steroid injection for De Quervain's tendonitis, which provided relief of pain, but the swelling persisted. Within one week, the patient also gradually developed diffuse severe joint pains in the right wrist and both elbows, shoulders, and hips. She was started on a methylprednisolone taper for six days and reported a 50% improvement in pain, but the severe joint pains recurred when she finished the regimen. After completing the methylprednisolone taper, her CRP level was slightly elevated at 9.8 mg/L, as shown in Table 1. Her antinuclear antibody and rheumatoid factor tests were negative. She was then treated with prednisone 30 mg daily, methotrexate 15 mg once weekly, and celecoxib. However, after four months of treatment, methotrexate and celecoxib were ineffective, and the patient could not reduce her prednisone dose.

**Table 1:** Patient A laboratory tests mg/L: milligrams per liter; mm/hr: millimeters per hour

	Reference range	Prior to starting upadacitinib treatment	6 months after starting upadacitinib treatment	11 months after starting upadacitinib treatment
C-reactive protein (CRP) (mg/L)	<5	9.8	<3	<1
Erythrocyte sedimentation rate (ESR) (mm/hr)	0-30	14	4	2
Antinuclear antibody (ANA)	≤1:40	<1:40	-	-
Rheumatoid factor (RF)	Negative	Negative	-	-
HLA-B27	Negative	Negative	-	-

In August 2023, the patient continued to have diffuse joint pains and was started on upadacitinib 15 mg daily while continuing low-dose daily prednisone. She reports joint pains resolved within two weeks of starting upadacitinib. Within six months of treatment, the CRP level normalized, as shown in Table 1. After eight months, the patient was able to discontinue prednisone treatment. Since starting upadacitinib, the patient was counseled on HZV, but she deferred the zoster vaccine. She did develop two episodes of HZV reactivation throughout the treatment course, but this was treated effectively with valacyclovir at the onset of symptoms, and the patient continues to take valacyclovir 1000 mg daily as prophylaxis.

Patient B is a 45-year-old female with a medical history significant for chronic sinusitis, asthma, Grave’s thyroid disease treated with thyroidectomy, tachycardia, postural orthostatic tachycardia syndrome (POTS), Von Willebrand's deficiency, and hysterectomy. In January 2022, she received a third dose of the COVID-19 vaccine. Approximately two weeks later, she developed diffuse body swelling, joint pains, and rashes. She was treated with a methylprednisolone taper for six days, and the symptoms resolved.

In September 2023, the patient contracted her fifth COVID-19 infection. Following each infection, the patient developed diffuse symmetric arthralgias without joint swelling. After treating each flare with glucocorticoids or naproxen, the joint pains gradually improved over a period of one month but returned when triggered by another COVID-19 infection. When the patient presented to the clinic in October 2023, on a physical examination, the patient had several tender joints in the right wrist, right hand, and bilateral feet, but no synovitis was noted. The patient was found to have elevated CRP at 17 mg/L, a slightly elevated erythrocyte sedimentation rate, and several slightly elevated cytokines: IL-4, IL-5, IL-10, IL-13, and IL-17, as shown in Table 2. The most elevated cytokine was IFN-gamma at 15.2 pg/mL. Her antinuclear antibody, rheumatoid factor, and anti-cyclic citrullinated peptide antibody tests were negative.

**Table 2:** Patient B laboratory tests mg/L: milligrams per liter; mm/hr: millimeters per hour; pg/mL: picograms per milliliter; IL: interleukin; IFN: interferon

	Reference range	Prior to starting upadacitinib treatment	2 weeks after starting upadacitinib treatment	2.5 months after starting upadacitinib treatment	7 months after starting upadacitinib treatment	10 months after starting upadacitinib treatment
C-reactive protein (CRP) (mg/L)	0-10	17	20	7	3	2
Erythrocyte sedimentation rate (ESR) (mm/hr)	0-32	27	14	7	3	2
IL-4 (pg/mL)	≤2.2	3.8	-	≤2.2	-	-
IL-5 (pg/mL)	≤2.1	2.7	-	≤2.1	-	-
IL-10 (pg/mL)	≤2.8	6.6	-	≤2.8	-	-
IL-13 (pg/mL)	≤2.3	5.2	-	≤1.7	-	-
IL-17 (pg/mL)	≤1.4	2.6	-	≤1.4	-	-
IFN-gamma (pg/mL)	4.2	15.2	-	6.3	-	-
Antinuclear antibody (ANA)	<1:80	Negative	-	-	-	-
Rheumatoid factor (RF) IgM (Immunoglobulin M)	<3.5	3.1	-	-	-	-
Rheumatoid factor (RF) IgA (Immunoglobulin A)	<14	1.4	-	-	-	-
Anti-cyclic citrullinated peptide antibody (CCP) IgG	<7	1.5	-	-	-	-
HLA-B27	Negative	Negative	-	-	-	-

The patient was started on upadacitinib 15 mg daily and received the HZV vaccine at the time of starting treatment. After 14 days of treatment, she reported a 90% improvement in joint pains. However, her CRP level remained elevated at 20 mg/L. Within 2.5 months of treatment, her pain completely resolved. Her CRP and several of the previously elevated cytokines normalized. The IFN-gamma level was also lowered to 6.3 pg/mL. By this point, on the physical examination, the patient had just one tender joint, in the right hand. She continues to take upadacitinib 15 mg daily and reports regaining function of joints and resolution of joint pains.

## Discussion

These two cases demonstrate the therapeutic effect of upadacitinib in treating post-COVID-19 inflammatory arthritis, which is increasingly recognized as a distinct autoimmune condition following COVID-19 vaccination and/or viral infection. Patients A and B presented with diffuse joint pains and elevated CRP levels following COVID-19 infections. While it appeared that the COVID-19 infection had resolved, the patients continued to experience arthralgias for months afterward. Notably, neither patient had autoimmune serologic abnormalities (antinuclear antibody, rheumatoid factor, cyclic citrullinated peptide), nor was there synovitis of multiple joints; therefore, they did not meet the criteria for diagnosis of antinuclear antibody-associated disease (such as lupus) or rheumatoid arthritis. This makes it more likely that the patients were dealing with a novel phenomenon of post-COVID-19 inflammatory arthritis that was mediated by a pathology different from those of lupus or RA.

Of note, both patients also reported joint pains after COVID-19 vaccination. This suggests that the SARS-CoV-2 antigen, whether through vaccination or viral infection, may trigger an autoimmune inflammatory arthritis in some individuals. One hypothesis suggests a mechanism of molecular mimicry, since components from the COVID-19 vaccine or pathogenic proteins from COVID-19 infections closely resemble human proteins [10,11]. In addition, prolonged immune activation from COVID-19 infection could contribute to the autoimmune pathogenesis. The vaccines may have triggered a new autoimmune arthritis, which was further exacerbated by the impact of COVID-19 infection on the immune system [12].

The lack of efficacy of methotrexate for patient A and both patients’ inability to discontinue steroid treatment without a recurrence of arthralgias demonstrate the utility of upadacitinib in treating their inflammatory arthritis. Upadacitinib is a JAK inhibitor that is used as a steroid-sparing agent in treating autoimmune diseases, including rheumatoid arthritis, psoriatic arthritis, and ankylosing spondylitis [5]. It has become a preferred alternative to glucocorticoids and disease-modifying anti-rheumatic drugs (DMARDs) such as methotrexate due to the relatively lower-risk side effect profile and rapid improvement in symptoms, making it a more ideal long-term solution for treating chronic autoimmune conditions. The specific mechanisms of JAK inhibitors on inflammatory cytokine pathways enable the suppression of autoimmune inflammation while minimizing the patient’s exposure to the risks of broader immunosuppression. In the two cases presented, treatment with upadacitinib provided significant improvement in joint pains within two weeks and normalization of CRP levels. For patient B, the normalization of her cytokine panel correlated with her improvement in symptoms following treatment, suggesting that these inflammation pathways were likely directly inhibited by upadacitinib.

Both patients tolerated upadacitinib well and achieved remission. However, it is important to acknowledge that treatment with upadacitinib should be in conjunction with close patient monitoring for side effects and adverse reactions [7]. When patient A developed HSV reactivation, her disease remained relatively mild due to the rapid implementation of antiviral treatment. Additionally, other adverse effects, such as blood counts, liver enzymes, and renal function, were monitored at least once every three months for these patients to ensure tolerability and safety.

## Conclusions

Patients with post-COVID-19 inflammatory arthritis should be considered for treatment with JAK inhibitors. The two patients highlighted in this case presented with arthralgias, arthritis, and elevated CRP levels following COVID-19 infections. The pathogenesis of this inflammatory arthritis is hypothesized to be due to molecular mimicry, due to the similarity in molecular structures of COVID-19 vaccine components, pathogenic proteins, and human proteins. The treatments in this case show the utility of upadacitinib in reducing synovitis and mediating acute phase reactants for those with post-COVID-19 inflammatory arthritis.
